# Unique Case of Cervical Split Cord Malformation Type II and Klippel-Feil Syndrome in a Male Patient Symptomatically Treated With Ultrasound-Guided Botulinum Toxin Injection

**DOI:** 10.7759/cureus.69531

**Published:** 2024-09-16

**Authors:** Mustafa Ali, Bogomil Iliev, Yavor Enchev, Georgi Boshev, Alkim Demirci

**Affiliations:** 1 Department of Neurosurgery and ENT Diseases, Division of Neurosurgery, Medical University of Varna, Varna, BGR; 2 Neurosurgery, University Hospital “St. Marina”, Varna, BGR

**Keywords:** botulinum injection, congenital spine deformity, klippel-feil syndrome, split cord malformation, torticollis

## Abstract

The neural tube abnormality known as split cord malformation (SCM) is characterized by longitudinally separated functional hemicords. SCM is the result of a single basic ontogenetic error and may be associated with other anomalies. One such anomaly is Klippel-Feil syndrome (KFS), which is characterized by abnormal fusion of two or more cervical vertebrae. We present the case of a 15-year-old boy with a history of mandibular deformity, neck pain, and stiffness. Diagnostic imaging revealed type II cervical SCM, KFS, C1 vertebral arch anomaly, thoracic syringomyelia, and S1 paraspinous cleft. He was treated symptomatically with ultrasound-guided intramuscular injection of botulinum toxin (BT). Follow-ups showed that the treatment had a good effect on pain and stiffness, and an improvement in head posture was also achieved. Seven months after the first injection, a second injection was performed because the effect of BT diminished. In this report, we present the first case of cervical SCM type II associated with KFS treated symptomatically with BT injection. This is also the first reported case in a male patient; only 10 cases with both anomalies have been published in PubMed, and all of these cases are in females.

## Introduction

Split cord malformation (SCM) is a congenital abnormality of the neural tube characterized by longitudinally separated functional hemicords [1]. In their unified theory, Pang et al. [2] explain that all SCMs result from a basic ontogenetic error that leads to the formation of a midline endomesenchymal tract. The lumbar region is most commonly affected (46-55% of cases), while the cervical region is affected in only 1-3% of cases [3]. Pang et al. [2] classified SCM into two types. In recent years, some authors have reported rare cases with features of both types of SCM, i.e. mixed, and proposed to define it as type 1.5 [1]. Musculoskeletal abnormalities, tethered cord, low-lying filum, syringomyelia, spina bifida, overlying skin changes, and other conditions may be associated with SCM [4]. One of these abnormalities is Klippel-Feil syndrome (KFS), which is characterized by abnormal fusion of two or more cervical vertebrae [3].

## Case presentation

Patient history

A 15-year-old boy presented with a six-month history of neck pain and stiffness aggravated by prolonged standing. In the past year, his parents noticed a deformity of the lower jaw and a tilting of the head to the left side. He was diagnosed with pectus excavatum at the age of 8 and scoliosis at the age of 10.

Clinical examination

The patient had facial asymmetry, mandibular deviation, and a maxillary canted occlusal plane. He had a low hairline, a short and wide neck with left side torticollis. The spine was scoliotic. In addition, there was pectus excavatum. Neurological status was normal.

Image findings

**Figure 1:**
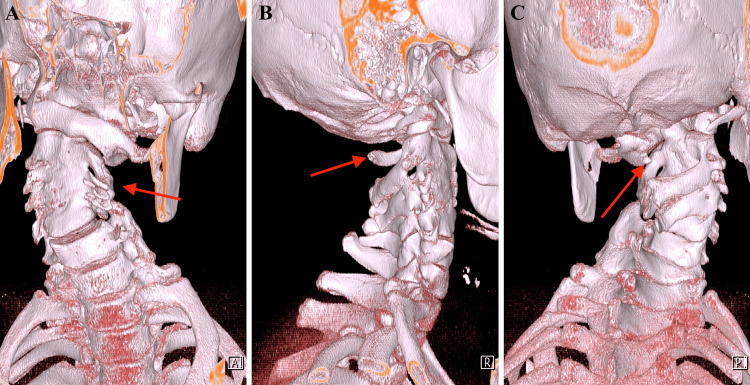
3D reconstruction of the CT scan CT, computed tomography; 3D, three-dimensional (A) Cervical fusion from C2 to C5 (red arrow). (B,C) Anomaly of the C1 vertebral arch (red arrow).

**Figure 2:**
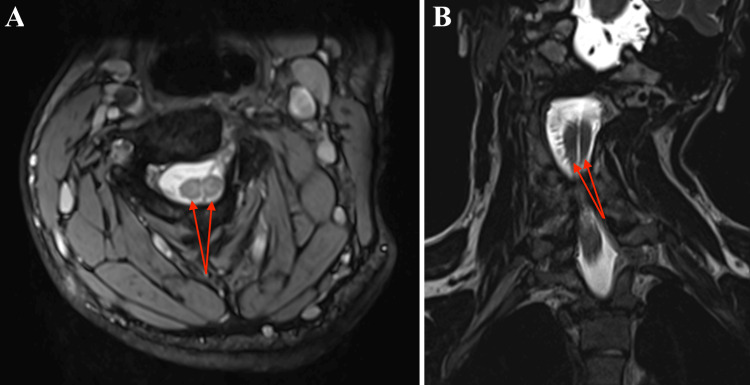
Cervical spine MRI MRI, magnetic resonance imaging (A) The axial plane of the MRI showing two hemicords within a single dural sac (red arrows). (B) The coronal plane of the MRI showing two hemicords (red arrows).

**Figure 3:**
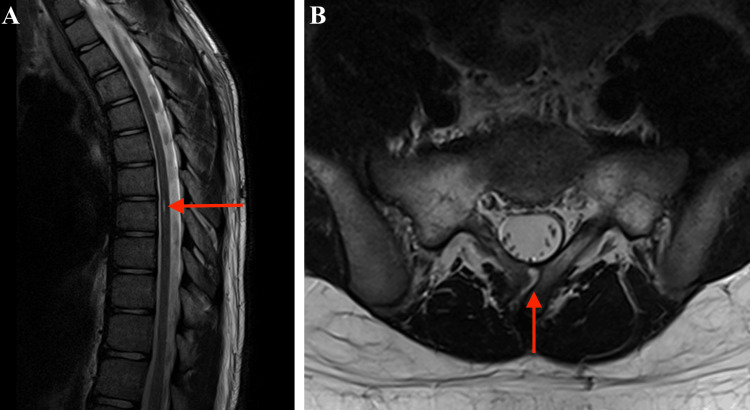
Thoracolumbar spine MRI MRI, magnetic resonance imaging (A) The sagittal plane of the MRI showing a syrinx at the level of the Th8 vertebra (red arrow). (B) The axial plane of the MRI showing a paraspinous cleft of the S1 vertebra on the right side (red arrow).

Magnetic resonance imaging (MRI) and computed tomography (CT) revealed an anomaly of the C1 vertebral arch and cervical fusion from C2 to C5 (Figure 1). At the same level, the spinal cord was divided into two segments within a single dural sac; the hemicords were not separated by a bony septum (Figure 2). A thoracolumbar MRI showed a syrinx at the level of the Th8 vertebra and a paraspinous cleft of the S1 vertebra on the right side (Figure 3). The tethered cord was absent.

Treatment and the patient outcome

The patient was treated with ultrasound-guided intramuscular injection of 100 international units (IU) of botulinum toxin (BT) type A into four muscle groups (Table 1). The results of the one-, three-, and five-month follow-up showed that the symptomatic treatment had a good effect on pain and stiffness, and an improvement in head posture was also achieved. Seven months after the BT injection, the effect of the treatment was diminished, and a second injection was performed.

**Table 1:** Ultrasound-guided intramuscular injection of botulinum toxin IU, international units

Muscles	Quantity injection sites per side	Right	Left
Levator scapulae	2	20 IU	-
Sternocleidomastoid	2	20 IU	20 IU
Descending part of the trapezius	2	-	20 IU
Anterior scalene	1	20 IU	-

## Discussion

**Figure 4:**
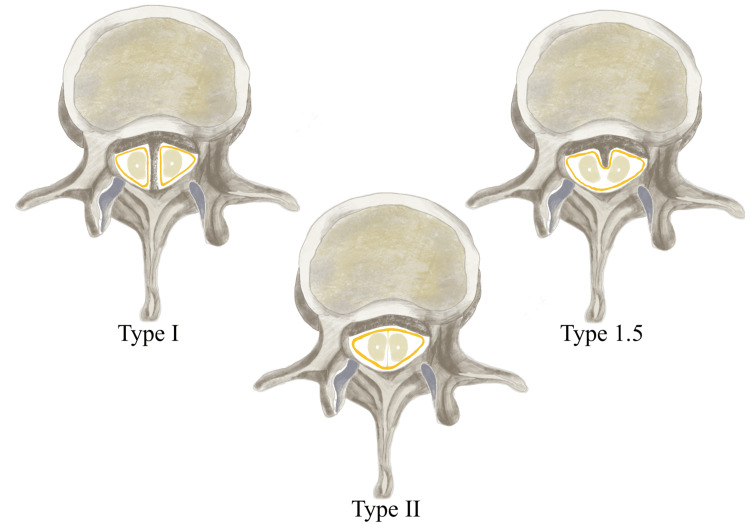
Hand-drawn the three types of split cord malformation The illustration is the author’s work with Procreate.

In their unified theory, Pang et al. [2] describe how all SCMs arise from a basic ontogenetic error that results in the formation of a midline endomesenchymal tract. The development of a type II or type I SCM depends on the timing of the appearance of this tract, which occurs either before or after 21 days of gestational age [2]. Pang et al. [2] classified SCM into two types based on the nature of the septum (osseocartilaginous or fibrous) and the dural tube (double or single) (Figure 4 and Table 2). In recent years, cases with characteristics of both types of SCM, i.e. mixed, have been published, suggesting a definition of type 1.5 [1] (Figure 4 and Table 2).

**Table 2:** Split cord malformation classification This table was created using the information from Sun et al. [1], Pang et al. [2], and Sreenivasan et al. [5].

	Type I (diastematomyelia)	Type 1.5	Type II (diplomyelia)
Hemicords	Double	Double	Double
Dural tube	Double	Single	Single
Septum	Rigid and osseocartilaginous	Rigid and osseocartilaginous	Nonrigid and fibrous
Incidence	45%	Only 15 reported cases	55%

The majority of SCM cases (46-55%) involve the lumbar spine, with only 1-3% affecting the cervical spine [3]. In their review, Sreenivasan et al. [5] found 71 reported cases of cervical SCM from 1889 to 2016. Approximately 50% of cervical SCM cases present clinically before the age of 10 years, and the male-to-female ratio is 2:5, but there is no explanation in the literature for this predominance [5]. To the best of our knowledge, only 10 cases of cervical SCM type II and KFS have been published in PubMed; all of these cases were in females, and our report is the first in a male with both disorders (Table 3).

**Table 3:** Reported cases with both cervical SCM type II and KFS SCM, split cord malformation; KFS, Klippel-Feil syndrome; F, female; M, male

Authors and year	Age of diagnosis	Sex	Clinical features	Image findings	Treatment
David et al. [6] 1996	5 months	F	Complaints: respiratory insufficiency	Unspecified cervical and thoracic fusions and mediastinal neurenteric cyst	Mediastinal neurenteric cyst removal
			Physical examination: short and deformed neck and limited cervical motion		
			Neurological examination: normal		
David et al. [6] 1996	7 years old	F	Complaints: neck pain	C1-C2 instability, unspecified cervical fusions, and occipital meningocele	Meningocele excision and occipito-cervical fixation
			Physical examination: head tilt and limited cervical motion		
			Neurological examination: normal		
David et al. [6] 1996	12 years old	F	Complaints: neck pain	Fusion and hemivertebrae C2-C7, occipital meningocele, and epidermal cyst	Cyst removal and ventriculoperitoneal shunt
			Physical examination: head tilt and limited cervical motion		
			Neurological examination: normal		
Ali et al. 2024 (present case)	14 years old	M	Complaints: neck pain and mandibular deformation	C1 arch anomaly C2-C5 fusion and syrinx at the level of the Th8 vertebra and S1 paraspinous cleft	Ultrasound-guided intramuscular injection of botulinum toxin
			Physical examination: head tilt, craniofacial dysmorphism, low posterior hairline, short neck, torticollis, pectus excavatum, and scoliosis		
			Neurological examination: normal		
David et al. [6] 1996	20 years old	F	Complaints: growing stopped at the age of 12	Unspecified cervical fusions, spina bifida, and meningocele	Follow-ups
			Physical examination: craniofacial dysmorphism, low posterior hairline, short neck, and limited cervical motion		
			Neurological examination: strabismus, bilateral conductive hearing loss, and gait ataxia		
Goacher and Lee [7] 2024	26 years old	F	Complaints: neck pain	C4-C7 fusion	No treatment has been carried out
			Physical examination: bimanual synkinesis		
			Neurological examination: normal		
David et al. [6] 1996	35 years old	F	Complaints: neck pain	Atlanto-occipital fusion, C2-C3 fusion, and spina bifida	Conservative pain management
			Physical examination: low posterior hairline, short neck, and limited cervical motion		
			Neurological examination: normal		
David et al. [6] 1996	36 years old	F	Complaints: occipital headache, dizzy spells, and blurred vision	C2-C6 fusion	Local anesthetic injection to the occipital nerve
			Physical examination: low posterior hairline, short neck, limited cervical motion, and short stature		
			Neurological examination: left temporal visual field defect, spastic quadriparesis		
David et al. [6] 1996	49 years old	F	Complaints: occipital pain, neck pain, right arm paraesthesia and weakness, and weakness of the legs	Atlanto-occipital fusion and C2-C7 fusion	No treatment has been carried out
			Physical examination: low posterior hairline, short neck, and limited cervical motion		
			Neurological examination: bilateral abducens nerve palsy, mild bilateral sensorineural hearing loss, weakness of right triceps, weakness of both hands, spastic paraparesis of low extremities, and diminished sensation of light touch between C6 and T1 dermatome in the right		
Sreenivasan et al. [3] 2020	55 years old	F	Complaints: neck pain and right arm pain	C2-C3 and C4-C7 fusions and agenesis of the posterior elements of C4 and C5	Nonsteroidal anti-inflammatory drugs, myorelaxants, gabapentin, physical therapy, ultrasound-guided pain medicaments injection of the superficial cervical plexus, and suprascapular and greater occipital nerves
			Physical examination: tenderness at the cervical spine, shoulder, and right intercostal region and limited cervical and lumbar motion		
			Neurological examination: bilateral patellar hyperreflexia and positive Hoffmann's sign on the right		
Lopez-Vicchi et al. [8] 2015	61 years old	F	Complaints: chronic headache	C1-C4 fusion	No treatment has been carried out
			Physical examination: head tilt, low posterior hairline, and short neck		
			Neurological examination: unilateral sensorineural hearing loss		

SCM may be associated with other conditions like musculoskeletal abnormalities, tethered cord, low-lying filum, syringomyelia, spina bifida, and cutaneous stigmata [4]. Cutaneous stigmata such as hypertrichosis, naevi, capillary hemangiomas, dimples, dermal sinus, and lipomatous swellings are seen in 60% of cases [5,9]. Spina bifida is seen in 18.9% of patients, tethered cord in 47.2%, low-lying filum (conus below L2) in 48.8%, and syringomyelia in 22% [9]. Musculoskeletal abnormalities are one of the most common abnormalities with scoliosis seen in 40% of SCM cases, hemivertebra in 5.9%, block vertebra and butterfly vertebra in 4%, rib abnormalities in 10%, and very rarely KFS [9]. This group of abnormalities also includes lower limb anomalies like congenital talipes equinovarus seen in 16.5%, congenital hip dislocation in 1.6%, flat foot in 4.3%, and thinning/shortening of limbs in 13% [9]. Our patient had the classic triad (low posterior hairline, short neck, and limited cervical mobility) for KFS, and this triad was seen in four of 10 similar cases (Table 3). A syrinx at the level of the Th8 vertebra and paraspinous cleft of the S1 was found as an additional imaging finding (Figure 3).

Type II SCM is usually diagnosed at an average age of 8 years [10]. Neurological symptoms in patients with type II SCM do not change significantly throughout life unless there are associated abnormalities such as a hemivertebrae or scoliosis with a Cobb angle less than 40° [11]. In type II SCM, pathologic features such as the spinal septum and adhesions are absent, which explains why many adult patients are very active [11]. Hang et al. [10] reported no improvement or worsening of neurological symptoms in operated patients with type II SCM; they recommended avoiding surgery in type II SCM and keeping the patients under observation. In reviewing the literature, we found that similar cases were treated conservatively, and when they underwent surgery, the cause was another associated condition (Table 3). Our patient was treated with ultrasound-guided intramuscular injection of BT. The one, three, and five-month follow-up results showed that symptomatic treatment had a good effect on pain and stiffness, and the head posture also improved. After seven months, the effect of injections diminished, and reinjection of BT was necessary. We must consider that the results of BT injection may not be as good as in functional torticollis if there are anatomical changes in the structures (vertebrae, spinal cord, nerve roots, muscles, etc.). If necessary, reinjection of BT can be discussed 10-12 weeks after the prior injection.

## Conclusions

SCMs are very rare in the cervical region. SCM may be associated with multiple anomalies, such as KFS. Associated abnormalities may influence the progression of the disease and must be considered when planning treatment methods. In this report, we present the first male patient with cervical SCM type II and KFS. In patients with both abnormalities, good results can be achieved with ultrasound-guided intramuscular injection of BT. We must consider that the results of BT injection may not be as good if there are anatomical changes in the structures (vertebrae, spinal cord, nerve roots, muscles, etc.). If necessary, reinjection of BT can be performed.
